# Can the MOLES acronym and scoring system improve the management of patients with melanocytic choroidal tumours?

**DOI:** 10.1038/s41433-022-02143-x

**Published:** 2022-06-28

**Authors:** Bertil E. Damato

**Affiliations:** Nuffield Laboratory of Ophthalmology, Department of Clinical Neurosciences, University of Oxford, and Ocular Oncology Service, Moorfields Eye Hospital, London, UK

**Keywords:** Physical examination, Health care

## Abstract

It can be difficult for practitioners to determine the likelihood of malignancy in melanocytic choroidal tumours. This author has therefore devised the MOLES acronym to highlight the most informative clinical features, which comprise mushroom shape, orange pigment, large size, enlargement, and subretinal fluid. Each of these is scored 0 if absent, 1 if subtle or uncertain, and 2 if present. Tumours are categorised as ‘common naevus’, ‘low-risk naevus’, ‘high-risk naevus’ and ‘probable melanoma’ according to whether the sum of these five scores is 0, 1, 2 or 3 or more, respectively. Tentative recommendations, subject to future studies, include: review of ‘common naevi’ by a community optometrist whenever the patient attends for another reason, such as a two-yearly ‘check-up’ (i.e., ‘self-care’); non-urgent referral of patients with ‘low-risk naevi’ or ‘high-risk naevi’ to an ophthalmologist to plan long-term surveillance (i.e., determining the frequency of assessments and whether these should be undertaken by an ophthalmologist or a community optometrist); and urgent referral of patients with a MOLES score >2 (i.e., ‘probable melanoma’) to an ophthalmologist for immediate referral to an ocular oncologist if a suspicion of malignancy is confirmed. The MOLES system does not require assessment of internal acoustic reflectivity by ultrasonography. MOLES scores correlate well with diagnosis of choroidal naevi and melanomas by ocular oncologists; however, further evaluation of this aid in routine optometric practice and other situations is needed. MOLES should prevent unnecessary referral of patients with naevi for second opinion and non-essential monitoring of these patients at hospital eye services.

## Abstract (zh)

临床工作者很难确定脉络膜黑色素瘤的恶性程度。因此, 本文作者设计了MOLES首字母缩略词来突出最具价值的临床特征, 包括蘑菇形态, 橙色色素, 大瘤体, 血管扩张和视网膜下积液。如果不存在上述临床特征, 则评分为0, 如果有细微临床表现或不确定, 则评分为1, 如果存在上述临床特征, 则评分为2。根据这5个评分的总和是否分别为0、1、2或3或以上, 我们将肿瘤分类为“常见痣”、“低风险痣”、“高风险痣”和“可能的黑色素瘤”。

针对未来的研究, 我们暂定的建议包括: 当病人其他原因就诊时, 应由社区验光师复查“普通痣”, 如病人两年一次的“检查” (即“自我保健”) ; 将患有“低风险痣”或“高风险痣”的患者非紧急转诊给眼科医生以计划长期监测 (即确定评估的频率以及决定是否应由眼科医生或社区验光师进行评估) ; 如果确认怀疑有恶性肿瘤, 则将MOLES评分 > 2 (即“可能的黑色素瘤”) 的患者紧急转诊给眼科医生, 以便立即转诊至眼部肿瘤科医生处。MOLES 系统不需要通过超声检查评估内部声学反射率。 MOLES 评分对眼部肿瘤专家对脉络膜痣和黑色素瘤的诊断密切相关; 然而, MOLES评分对于诊断的辅助性需要在常规视光学门诊和其他情况下进行进一步调研。MOLES 旨在防止痣病人不必要的二次转诊以及眼科医疗服务机构对这些病人进行不必要的监测。

## Introduction

Early treatment of choroidal melanoma optimises any opportunities for conserving vision and the eye and may prevent metastatic disease in some patients [1–3]. It can be difficult to distinguish small choroidal melanomas from naevi [4]. Community optometrists are therefore referring large numbers of patients with choroidal naevi to hospital eye services (Damato et al., Oxford Eye Hospital audit, unpublished data). Many of these patients are then monitored for months or years at a hospital eye service and some are referred to a tertiary-referral ocular oncology centre for diagnosis. Conversely, patients with melanoma may experience long delays in treatment, because their tumour is monitored for growth [5, 6]. The problem is compounded by the 5-8% prevalence of choroidal naevi, few of which ever show malignant growth [7–10].

The tendency to refer patients for expert opinion can be expensive for patients, who may incur travel expenses, loss of income and other costs and who may also be stressed by the possibility of eye cancer and the risk of catching Covid-19 while travelling to and from the hospital eye service and in the clinic itself. Non-essential specialist care also diverts healthcare resources from patients in greater need, lengthening hospital waiting lists and overburdening photography units, ultrasonography departments, and other services, possibly delaying the diagnosis and treatment of patients with melanoma and other serious conditions requiring urgent treatment, especially during the Covid-19 pandemic and its aftermath [11].

The author has developed the MOLES acronym and scoring system, to help non-experts estimate the likelihood of malignancy in melanocytic choroidal tumours and to manage patients accordingly. The aims of this article are to describe MOLES and to discuss how it could improve the management of patients with these lesions.

### MOLES acronym

MOLES stands for: mushroom shape, orange pigment, large size, enlargement, and subretinal fluid (SRF), all of which are well known to be indicators of malignancy.

#### Mushroom shape

Choroidal melanomas develop a mushroom (‘collar-stud’) shape when they perforate the retinal pigment epithelium (RPE) so that Bruch’s membrane obstructs venous outflow from the intra-retinal part of the tumour, which becomes oedematous and swollen. On ophthalmoscopy and colour photography, the exposed, apical part of the tumour shows dilated and tortuous tumour vessels (‘double circulation’). The base of the tumour is usually grey, even with amelanotic melanomas, because of multilayering of the RPE, which may also show clumps of orange pigment [12]. On ultrasonography (US), the intra-retinal part of the tumour shows high internal acoustic reflectivity, because of the interstitial oedema, whereas the basal part of the tumour, within the choroid, shows low reflectivity, because the tumour cells are packed close together.

Perforation of Bruch’s membrane and RPE by a naevus is rare and usually minimal, without the development of a mushroom shape [13].

#### Orange pigment

**Fig. 1:**
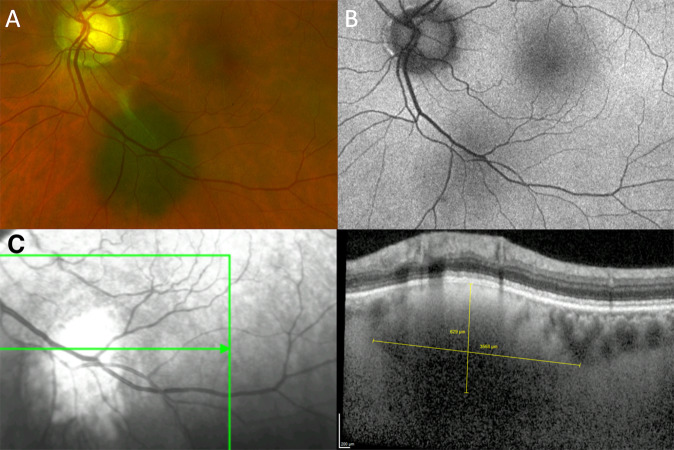
Choroidal naevus (MOLES score = 00000 = ‘common naevus’). **A** Colour photograph showing the naevus infero-temporal to the left optic disc, (**B**) FAF, showing no hyper-autofluorescent lipofuscin, and (**C**) OCT, showing a normal RPE and retina, with no lipofuscin or subretinal fluid. This scan also shows how it is possible to measure tumour diameter and, if the internal scleral surface can be identified, the tumour thickness.

**Fig. 2:**
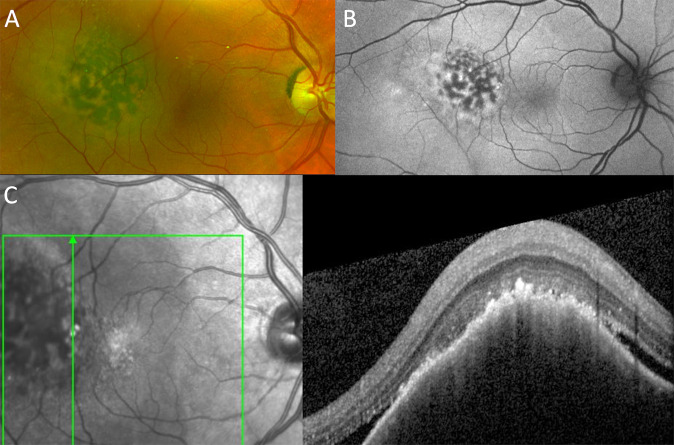
Choroidal melanoma (MOLES score = 02102 = 5 = ‘probable melanoma’). **A** Colour photograph, showing a dome-shaped choroidal melanoma temporal to the right fovea, with clumps of confluent orange pigment, (**B**) FAF, showing the lipofuscin to be hyper-autofluorescent, and (**C**) OCT, showing lipofuscin clumps and subretinal fluid.

Lipofuscin is a waste product from the retinal receptors, which is normally removed by the RPE [14]. When this function is disrupted by an underlying tumour, lipofuscin accumulates as dusting if mild and as clumps if severe. This pigment is normally minimal or absent over choroidal naevi (Fig. 1). It is usually present over choroidal melanomas (Fig. 2) On ophthalmoscopy and colour photography, lipofuscin is orange over dark tumours (hence the term ‘orange pigment’) (Fig. 2). On FAF imaging, lipofuscin is brightly autofluorescent (Fig. 2). On optical coherence tomography (OCT), the lipofuscin accumulates on the superficial (inner) surface of the RPE (Fig. 2). Lipofuscin can appear brown or black over amelanotic tumours. In some cases, fundus autofluorescence may reveal lipofuscin that has desquamated from the tumour surface, forming deposits in subretinal fluid inferior to the tumour, occasionally forming a ‘pseudo-hypopyon’. Lipofuscin may be absent over a melanoma if the tumour is very small with minimal thickening. It is the author’s impression that lipofuscin is less likely to develop pre-equatorially and that it tends to disappear when tumour thickness increases beyond 2–3 mm, possibly when retinal receptors atrophy so that lipofuscin is no longer produced. Orange pigment can also accumulate over choroidal haemangiomas and metastases. In some cases, the lipofuscin can be difficult to see without FAF. Some choroidal naevi have what appears to be confluent orange pigment, which proves to be transparent RPE over an amelanotic tumour. Unlike lipofuscin, drusen show minimal autofluorescence and develop between the RPE and Bruch’s membrane.

#### Large size

There is much size overlap between melanomas and naevi. A study by Augsburger et al. found that there are approximately 70, 10 and 3 naevi for every choroidal melanoma in the basal diameter ranges of 5–6 mm, 6–7 mm, 7–8 mm, respectively [15]. Thin tumours tend to have tapering margins, so that colour photography may define tumour extent more accurately than US [16]. Despite some variation in optic disc diameter basal tumour dimensions of such lesions can be measured more accurately by ophthalmoscopy or colour photography, considering one horizontal disc diameter (DD) to be equivalent to 1.5 mm. In some cases, green-free photographs can show the tumour extent more readily than colour photographs. Ideally, a ruler or callipers should be used for these measurements, because visual estimates can be inaccurate. OCT can be useful in some cases (Fig. 1).

Augsburger et al. also found that there are approximately 125, 25 and 5 choroidal naevi for every melanoma in the thickness range of 1.5–2 mm, 2–2.5 mm, 2.5–3 mm, respectively [15]. The thickness of small, posterior lesions can be measured by performing OCT if the internal scleral surface can be seen (Fig. 1). With halo naevi, this technique may show a flat anterior surface with posterior bowing of the internal scleral surface, which may be missed with ultrasonography [17]. Nevertheless, ultrasonography may be useful for measuring tumour thickness with posterior tumours when OCT is not possible because of large tumour size and with peripheral tumours if they appear bulky. The callipers should be placed at the inner scleral surface and the tumour apex, excluding sclera and sensory retina. Tumour thickness measurements obtained by ultrasonography are significantly greater than those obtained with OCT [18].

#### Enlargement

Choroidal naevi rarely enlarge after the second decade of life. Mashayekhi et al. reported a median increase in diameter of 0.06 mm per year (range, 0.01–0.36) in tumours with a median diameter of 5 mm (range, 0.5–14) [19]. In contrast, small melanomas tend to enlarge at a rate of 0.25 mm per year [20]. However, this author believes that the most sensitive way of detecting enlargement is by assessing distances between tumour margins and adjacent landmarks. The entire tumour margin should be assessed because lateral extension may not occur in the same axis of the largest basal diameter. Furthermore, photographic distortion can give a false impression of growth [21]. According to Jouhi et al., the basal diameter of melanomas tends to increase by approximately 34% per year as compared to 1% in naevi [20].

Because of measurement variation with ultrasonography, apparent increase in tumour thickness is considered significant only if it exceeds 0.5 mm and is confirmed by repeated testing. Ultrasonography can give a false impression of growth if later thickness measurements include sclera and/or retina or if comparisons are made with previous OCT, which tends to give smaller values. It is rare for tumours to grow thicker without also becoming wider so that US is unlikely to reveal growth in the absence of other clinical signs of progression [22]. Even without growth, some tumours develop increasing amounts of SRF, and/or orange pigment as well as RPE perforation, which should be regarded as indicating activity [22]. For these reasons, sequential colour photography is more sensitive than US for detecting tumour progression.

Some patients are referred urgently with what appears to be a common choroidal naevus because the lesion was not documented previously. Although some naevi appear in later life, a more common explanation is that the naevus was missed or not mentioned to the patient on previous examinations. Nevertheless, these patients need monitoring to exclude malignancy.

#### Subretinal fluid

Subretinal fluid accumulates when RPE function is disturbed by the underlying choroidal tumour. The retina is flat over common naevi, but some larger naevi can develop mild detachment, which is more extensive if caused by a choroidal neovascular membrane [23]. Intra-retinal cystoid oedema indicates chronicity and is not a sign of malignancy [24]. Neither is outer retinal atrophy inferior to the tumour, caused by previous or intermittent retinal detachment.

#### Features excluded from MOLES

Proximity to optic disc and absence of halo and/or drusen are excluded from MOLES because they show only weak associations with malignancy [25]. Visual symptoms are excluded because they are non-specific and because their cause is usually evident on examination. Low internal acoustic reflectivity (‘hollowness’) is excluded because it often cannot be assessed because of small tumour size, unavailability of sensitive US equipment, or lack of expertise. MOLES also excludes melanocytoma and congenital ocular melanocytosis, because of their known risk of malignant transformation, which indicates life-long monitoring of all patients [26, 27].

### MOLES scores

Each of the five MOLES items is given a score of 0 if absent, 1 if mild or uncertain, and 2 if present (Table 1).

**Table 1:** The MOLES acronym and scoring system for categorising melanocytic choroidal tumours according to likelihood of malignancy.

Risk factor	Severity	Score
Mushroom shape	Absent	0
	Unsure/Early growth through RPE	1
	Present, with overhang	2
Orange pigment	Absent	0
	Unsure/Trace (i.e., dusting)	1
	Confluent clumps	2
Large Size^a^	Thickness <1.0 mm (‘flat/minimal thickening’) and diameter <3 DD	0
	Thickness = 1.0–2.0 mm (‘subtle dome shape’) and/or diameter = 3–4 DD	1
	Thickness >2.0 mm (‘significant thickening’) and/or diameter >4 DD	2
Enlargement^b^	No growth or no previous ophthalmoscopy	0
	Unsure growth/‘new’ lesion not documented after previous ophthalmoscopy	1
	Definite growth or new tumour confirmed with sequential imaging	2
Subretinal fluid	Absent	0
	Trace (if minimal and detected only with OCT)	1
	Definite, seen without OCT, or extending beyond tumour margin	2
	Total score:	
	Tumour category^c^	

*DD* disc diameter (=1.5 mm).

^a^Ignore thickness if this cannot be measured.

^b^Assume enlargement has occurred if thickness >3 mm or diameter >5 DD and give this a score of 2.

^c^Categorise tumours according to total score: 0 = Common naevus; 1 = Low-risk naevus; 2 = High-risk naevus and 3 or more = Probable melanoma.

#### Mushroom shape

Mushroom shape is almost pathognomonic of melanoma. It is nevertheless given a score of only 2 to simplify the scoring system. This is possible because tumours that have this feature inevitably have a thickness exceeding 1 mm and, therefore, a score of at least 3, indicating probable malignancy. A tumour that has perforated RPE without forming a nodule with overhang (i.e., ‘incipient mushroom’) is given a score of 1.

#### Orange pigment

Lipofuscin is given a score of 1 if there is only fine dusting and 2 if forming confluent clumps (Fig. 2). If the examiner is unable distinguish between drusen and clumps of lipofuscin, this indicator is given a score of 1.

#### Large size

The 4 DD cut-off for giving largest basal tumour diameter a score of 2 is guided by the study of Augsburger et al., which suggests that approximately 10% of tumours measuring 6 to 7 mm in diameter are malignant. Similarly, 2 mm is selected as the cut-off for scoring tumour thickness as 2 because according to Augsburger et al. about 4% of tumours measuring 2.0–2.5 mm are malignant. The presence or absence of other indicators of malignancy should increase the sensitivity and specificity of MOLES when determining whether the tumour is likely to be a melanoma.

Some practitioners have expressed concerns to the author about being unable to measure thickness. By categorising tumour size according to diameter and/or thickness, MOLES enables examiners to ignore thickness if they cannot measure this. In a study by Al Harby et al., tumour thickness influenced the MOLES score in only 6/222 (2.7%) tumours [28]. This finding suggests that tumour size can be scored according to basal diameter alone if OCT is not possible because of peripheral tumour location or if this imaging equipment is not available. If the tumour is located within the range of OCT but too thick for accurate measurement with such imaging, this would indicate a thickness exceeding 2 mm, which would have a MOLES score of 2.

#### Enlargement

Tumour enlargement is given a score of 2 if confirmed by sequential imaging and this is irrespective of the age of the patient because melanoma can develop before adulthood, albeit rarely, so that monitoring is required to ensure that tumour growth ceases [29, 30]. Although naevi can grow after the first two decades of life, such growth is unusual so that it would be prudent to monitor such lesions.

In the absence of other indicators of malignancy, newly discovered lesions are given a score of 0 if no ophthalmoscopy was ever performed previously, 1 if ophthalmoscopy was performed without photography, and 2 if imaging did not previously demonstrate the tumour. Monitoring of these lesions seems reasonable in view of a study by Jouhi et al., which suggests that metastatic disease from choroidal melanomas is highly unlikely if the tumour is less than 3.0 mm in diameter at the time of treatment [31].

In some cases, particularly when the tumour is large and peripheral, tumour size is the only indicator of malignancy, especially if only a colour photograph is assessed, as in a virtual clinic. To avoid such melanomas from being scored as a high-risk naevus, the author has recently revised MOLES so that enlargement is assumed to have occurred if the tumour diameter is greater than 5 DD or if the thickness exceeds 3 mm.

When treatment of a suspicious melanocytic choroidal tumour is delayed until progression is observed, it is essential to ensure that the patient fully understands the risks and benefits of this management and that informed consent is adequately documented [32].

#### Subretinal fluid

Retinal detachment is given a score of 0 if absent, 1 if evident only on OCT and/or US, and 2 if visible ophthalmoscopically or if the subretinal fluid extends beyond the tumour margins. In the absence of subretinal fluid, intra-retinal cystoid oedema is given a score of 0 because it is a sign of chronicity, as are RPE and outer retinal degeneration, which are caused by fluid previously gravitating from the tumour.

#### Total score

Tumours are categorised as ‘common naevus’, ‘low-risk naevus’, ‘high-risk naevus’ and ‘probable melanoma’ according to whether the total score is 0, 1, 2 or 3 or more, respectively (Table 1).

Tumours are succinctly described by a five-digit code composed of these five scores. For example, a tumour with a basal diameter of 4.5 DD, clumps of confluent orange pigment, traces of subretinal fluid on OCT, and confirmed growth on sequential colour photography would have a MOLES code of 02221.

The term ‘common’ is preferred to ‘typical’ because naevi can be atypical without showing any indicators of malignancy. Tumours with a score of 3 or more are termed ‘probable’ melanoma because non-experts may not feel confident about making a diagnosis of melanoma and differentiating this from metastasis, haemangioma, and other tumours.

#### Management recommendations

For patients in the UK, the author has made tentative recommendations, which are broadly in keeping with guidelines of the College of Optometrists, the Royal College of Ophthalmologists, and the National Health Service guidelines for the management of patients with suspected cancer [33–35]. These recommendations need to be evaluated by further studies, ideally performed independently in optometry and a wide variety of other situations.

Common naevi can be reassessed by community optometrists when the patient attends for any other reason, such as broken spectacles, etc. (i.e., ‘self-care’). Patients should be informed of any naevus and ideally provided with a photograph of the lesion to take to the optometrist or ophthalmologist at every visit.

Pending further audit, the MOLES protocol tentatively suggests that patients with low-risk naevi and high-risk naevi should be referred non-urgently to an ophthalmologist for multimodal imaging and plans for long-term surveillance, advising on the assessment frequency, imaging methods, and when monitoring can be undertaken by a community optometrist (e.g., immediately or if no tumour progression has been documented after a specified time).

Patients with probable melanoma should be referred urgently to an ophthalmologist following NHS England’s two-week-wait protocol for suspected cancer [36].

Some ophthalmologists are concerned that excessive numbers of patients with a MOLES score of 1 will be referred to a hospital eye service. In early 2020, the author and associates audited the referral of 179 patients with melanocytic choroidal tumours to a diagnostic oncology service at Oxford Eye Hospital between 1 September 2018 and 31 December 2019 (Damato et al., Oxford Eye Hospital audit, unpublished data). Low-risk naevi comprised 18% of all tumours. The remainder consisted of common naevi (94, 53%); high-risk naevi (34, 19%) and probable melanoma (18, 10%). Until audits confirm that optometrists can reliably score suspicious melanocytic choroidal tumours, it would seem prudent for suspicious naevi to be assessed by an ophthalmologist.

The author and associates have designed adult ocular oncology referral forms for Oxford Eye Hospital and Moorfields Eye Hospital, which include the MOLES scoresheet, tips on assessing clinical features, suggested management, and links to referral guidelines [37, 38]. These measures are designed to provide information at the point of need to avoid the referrer having to remember guidelines or to spend time consulting other sources of information.

Ocular oncologists are increasingly triaging new referrals to virtual clinics or video-/phone-clinics whenever possible, to avoid patients from attending a clinic in person unnecessarily. Such triage is possible only if all relevant images of the lesion are received together with the referral form and letter. If growth has been documented, the oldest and most recent images are required. Failure to submit adequate images of the lesion without providing a good reason may result in the referral being placed on hold while the images are requested. If adequate images of the lesion are not possible, some centres, such as Oxford Eye Hospital, are giving patients an appointment at the hospital’s photography unit (i.e, ‘imaging hub’) for imaging, which is reviewed by an ophthalmologist within one or two days.

Some ocular oncology centres are now accepting only tertiary referrals from consultant ophthalmologists, to avoid non-essential referrals to such specialised services. Optometrists are being advised to refer patients to an ophthalmologist (not an ocular oncologist) and to do so directly not via the general practitioner. An audit in 2012 showed that patients experienced a median delay of 44 days if referred by their optometrist to an ophthalmologist via their general practitioner as compared to 10 days if referred directly [5]. As general practitioners usually lack facilities for imaging the ocular fundus, patients presenting to them should be referred to an optometrist for full ocular examination, imaging and referral to an ophthalmologist if the MOLES score is greater than 0, with images of the lesion in question as mentioned above.

### Validation

Al Harby and associates at the Ocular Naevus Clinic of Moorfields Eye Hospital reviewed imaging of 222 melanocytic choroidal tumours and retrospectively gave these MOLES scores [28]. All 81 tumours diagnosed as melanoma by ocular oncologists were found to have a MOLES score of 3 or more (i.e., 100% sensitivity) whereas 135 out of 141 naevi had a MOLES score less than 3 (95.7% specificity). Of the six tumours with discordant diagnoses, four had a basal diameter exceeding 6 mm, all with SRF and/or lipofuscin, and two small tumours showed either significant SRF with traces of lipofuscin, or vice versa.

Roelofs et al. reviewed the imaging of 451 treated choroidal melanomas and retrospectively gave these MOLES scores [39]. Only one melanoma had a score less than 3; whether the discrepancy occurred because of a weakness of MOLES or whether another feature such as retinal detachment was not seen or documented is uncertain.

### Comparison with TFSOM-DIM system

The mnemonic, TFSOM-DIM (To-find-small-ocular-melanomas-doing-imaging) was developed by Shields et al. to highlight thickness greater than 2 mm, fluid under the retina, symptoms, orange pigment, melanoma hollow on ultrasonography, and diameter greater than 5 mm [40–42]. The Shields also developed a scoring system, based on the number of TFSOM-DIM findings, to predict future growth of the tumour, which is taken to indicate malignant transformation of a naevus. In contrast to TFSOM-DIM, MOLES uses growth as an indicator of malignancy.

Unlike TFSOM-DIM, MOLES avoids binary scoring, because non-experts may feel unable to decide whether a particular feature is present. Some authors are concerned that the intermediate score of 1 would result in over-referral of patients to a hospital eye service if optometrists decide to ‘play safe’. The author’s concern is that without a score of 1, such cautious optometrists will score uncertain findings as 2, so that tumours are categorised as ‘probable melanoma’, with the result that these patients are referred urgently instead of routinely. Unlike MOLES, TFSOM-DIM includes hollowness on US, which suggests that this system is designed for ophthalmologists having the skills and equipment to assess internal acoustic reflectivity of small choroidal tumours. To a large extent, MOLES and TFSOM-DIM are complementary, intended for non-experts and experts respectively.

### Clinical implications

MOLES was developed primarily to help optometrists refer patients to hospital eye services only if their tumour shows any suspicious clinical features and to follow the two-week-wait NHS protocol for suspected cancer only if the clinical features are sufficiently suspicious of malignancy. Various measures have been taken to enhance compliance with MOLES, which include: (a) a brochure with guidelines on detecting and scoring the MOLES indicators of malignancy, (b) referral forms with useful tips and links to informative websites, and (c) a publication in the optometric literature, similar to the present article [43]. The MOLES scoring system itself is designed to compensate for difficulties non-experts may encounter in detecting and scoring relevant clinical signs because of limited imaging equipment or experience.

The Oxford audit included assessment of patients with common naevi referred by ophthalmologists (Damato et al., Oxford Eye Hospital audit, unpublished data). There were 41 such patients, of whom 13 (32%) were referred after one hospital visit, 22 (54%) after 2–5 visits and 4 (10%) after 7–12 visits. In two (5%) patients, referred from other hospitals, the number of visits in those hospitals was unknown. Several patients had undergone repeated ultrasonography, and some also had angiography. The MOLES system would have encouraged the hospital ophthalmologists to discharge these patients with instructions on self-care.

The success of MOLES will depend greatly on the ability of its users to detect the clinical features of malignancy and to estimate or measure tumour dimensions reasonably accurately. Flanagan and associates performed a study in which 39 optometrists evaluated imaging of 25 melanocytic choroidal tumours. Using MOLES, these optometrists correctly identified 389 of 406 images of probable melanomas (95.8% sensitivity) and 331 of 516 images of choroidal naevi (64.1% specificity), erring on the side of caution [44]. The wider deployment of wide-angle fundus photography, optical coherence tomography and autofluorescence imaging in the community as well as hospital eye services should enhance the value of MOLES over time.

By excluding acoustic hollowness, MOLES avoids the need for all patients with melanocytic choroidal tumours to attend a hospital eye service for ultrasonography, thereby enhancing opportunities for tele-ophthalmology, as is already happening at Oxford Eye Hospital, Moorfields Eye Hospital, and a growing number of other centres.

### Scope for further research

Further studies are needed to audit the deployment of MOLES in routine optometric practice and to evaluate its impact on the quality of referrals to hospital eye services. It would be useful to determine how well optometrists’ scoring of each item correlates with an ophthalmologist’s assessment. This may identify any shortcomings in clinical practice that could be mitigated by further education or better imaging equipment. The findings of such studies may also indicate whether it is safe for optometrists in the community to monitor low-risk naevi without referring patients with these lesions to a hospital eye service.

There may be scope for evaluating the scoring system itself. For example, the scores arbitrarily given to orange pigment and subretinal fluid merit validation. Furthermore, it would be useful to investigate scoring the rate of tumour growth taking account of the tumour size.

Several research groups are developing artificial intelligence to diagnose ocular tumours, including choroidal naevi and melanomas. Ideally, these tools would be trained with images of tumours that are categorised according to their long-term outcomes [45]. However, it may take many years for sufficient data to be collected. In the meantime, it may be useful to train artificial intelligence with melanocytic choroidal tumours categorised using MOLES.

## Conclusions

Studies by ocular oncologists suggest that MOLES could help non-experts estimate the likelihood of malignancy in melanocytic choroidal tumours. Further studies are needed to evaluate this diagnostic aid in routine optometric practice and other clinical environments.

## Summary

### What was known before

- It can be difficult to distinguish small choroidal melanomas from naevi so that patients with naevi are referred unnecessarily to an ophthalmologist or ocular oncologist while those with melanoma experience delay in diagnosis and treatment.
- Suspicious features include mushroom (‘collar-stud’) shape, lipofuscin (i.e., ‘orange pigment’), large tumour size, tumour growth and presence of subretinal fluid.

### What this study adds

- The acronym MOLES has been devised to help non-experts remember the most informative indicators of malignancy (i.e., mushroom shape, orange pigment, large size, enlargement, and subretinal fluid).
- A system has been developed to score each of the five MOLES indicators as 0, 1 or 2 according to whether it is absent, uncertain/borderline, or present respectively.
- Tumours are categorised as ‘common naevus’, ‘low-risk naevus’, ‘high-risk naevus’ and ‘probable melanoma’ according to whether the sum total of the five item scores is 0, 1, 2 or 3 or more respectively (i.e., the 'MOLES Score').
- MOLES correlates well with diagnosis made by ocular oncologists, but needs to be evaluated when deployed by optometrists and ophthalmologists in a variety of clinical situations.

## References

[1] Hussain RN, Coupland SE, Kalirai H, Taktak AFG, Eleuteri A, Damato BE, et al. Small high-risk uveal melanomas have a lower mortality rate. Cancers (Basel). 2021;13:2267. 10.3390/cancers13092267 PMC8125943 34066842

[2] Damato BE, Heimann H, Kalirai H, Coupland SE (2014). Age, survival predictors, and metastatic death in patients with choroidal melanoma: tentative evidence of a therapeutic effect on survival. JAMA Ophthalmol.

[3] Callejo SA, Dopierala J, Coupland SE, Damato B (2011). Sudden growth of a choroidal melanoma and multiplex ligation-dependent probe amplification findings suggesting late transformation to monosomy 3 type. Arch Ophthalmol.

[4] Khan J, Damato BE (2007). Accuracy of choroidal melanoma diagnosis by general ophthalmologists: a prospective study. Eye.

[5] Damato EM, Damato BE. Detection and time to treatment of uveal melanoma in the United Kingdom: An Evaluation of 2384 Patients. Ophthalmology. 2012;119:1582–9. 10.1016/j.ophtha.2012.01.048 22503229

[6] Damato B (2001). Detection of uveal melanoma by optometrists in the United Kingdom. Ophthalmic physiological Opt: J Br Coll Ophthalmic Opticians.

[7] Sumich P, Mitchell P, Wang JJ (1998). Choroidal nevi in a white population: the Blue Mountains Eye Study. Arch Ophthalmol.

[8] Singh AD, Kalyani P, Topham A (2005). Estimating the risk of malignant transformation of a choroidal nevus. Ophthalmology.

[9] Kivela T, Eskelin S (2006). Transformation of nevus to melanoma. Ophthalmology.

[10] Qiu M, Shields CL (2015). Choroidal nevus in the united states adult population: racial disparities and associated factors in the national health and nutrition examination survey. Ophthalmology.

[11] Damato B (2020). Managing patients with choroidal melanoma in the COVID-19 era: a personal perspective. The. Br J Ophthalmol.

[12] Damato BE, Foulds WS (1990). Tumour-associated retinal pigment epitheliopathy. Eye.

[13] Weiss SJ, Stathopoulos C, Shields CL (2019). Choroidal nevus with retinal invasion in 8 cases. Ocul Oncol Pathol.

[14] Sepah YJ, Akhtar A, Sadiq MA, Hafeez Y, Nasir H, Perez B (2014). Fundus autofluorescence imaging: fundamentals and clinical relevance. Saudi J Ophthalmol.

[15] Augsburger JJ, Correa ZM, Trichopoulos N, Shaikh A (2008). Size overlap between benign melanocytic choroidal nevi and choroidal malignant melanomas. Investigative Ophthalmol Vis Sci.

[16] Pe’er J, Sancho C, Cantu J, Eilam S, Barzel I, Shulman M (2006). Measurement of choroidal melanoma basal diameter by wide-angle digital fundus camera: a comparison with ultrasound measurement. Ophthalmologica.

[17] Dolz-Marco R, Hasanreisoglu M, Shields JA, Shields CL (2015). Posterior scleral bowing with choroidal nevus on enhanced-depth imaging optical coherence tomography. JAMA Ophthalmol.

[18] Shields CL, Kaliki S, Rojanaporn D, Ferenczy SR, Shields JA (2012). Enhanced depth imaging optical coherence tomography of small choroidal melanoma: comparison with choroidal nevus. Arch Ophthalmol.

[19] Mashayekhi A, Siu S, Shields CL, Shields JA (2011). Slow enlargement of choroidal nevi: a long-term follow-up study. Ophthalmology.

[20] Jouhi S, Al-Jamal RT, Tall M, Eskelin S, Kivela TT. Presumed incipient choroidal melanoma: proposed diagnostic criteria and management. Br J Ophthalmol. 2021;bjophthalmol-2020-318658. 10.1136/bjophthalmol-2020-318658. Epub ahead of print. 10.1136/bjophthalmol-2020-318658 34666992

[21] Paul Brett J, Lake A, Downes S (2016). Colour imaging in the monitoring and documentation of choroidal naevi. Are Optomap colour images adequate for this purpose?. J Vis Commun Med.

[22] Roelofs KA, O’Day R, Al Harby L, Hay G, Arora AK, Cohen VML, et al. Detecting progression of melanocytic choroidal tumors by sequential imaging: is ultrasonography necessary? Cancers (Basel). 2020;12:1856. 10.3390/cancers12071856 PMC7408899 32664236

[23] Papastefanou VP, Nogueira V, Hay G, Andrews RM, Harris M, Cohen VM (2013). Choroidal naevi complicated by choroidal neovascular membrane and outer retinal tubulation. Br J Ophthalmol.

[24] Gass JD (1977). Problems in the differential diagnosis of choroidal nevi and malignant melanomas. The XXXIII Edward Jackson Memorial Lecture. Am J Ophthalmol.

[25] Shields CL, Maktabi AM, Jahnle E, Mashayekhi A, Lally SE, Shields JA (2010). Halo nevus of the choroid in 150 patients: the 2010 Henry van Dyke Lecture. Arch Ophthalmol.

[26] Singh AD, De Potter P, Fijal BA, Shields CL, Shields JA, Elston RC (1998). Lifetime prevalence of uveal melanoma in white patients with oculo(dermal) melanocytosis. Ophthalmology.

[27] Shields CL, Joffe, L, Shields, JA Melanocytoma of the optic disc. In: Schachat AP, editor. Ryan’s retina. Vol. 3. 6th ed. Edinburgh: Elsevier; 2018. p. 2484–9.

[28] Al Harby L, Sagoo MS, O’Day R, Hay G, Arora AK, Keane PA (2021). Distinguishing choroidal nevi from melanomas using the MOLES algorithm: evaluation in an ocular nevus clinic. Ocul Oncol Pathol..

[29] Al-Jamal RT, Cassoux N, Desjardins L, Damato B, Konstantinidis L, Coupland SE (2016). The pediatric choroidal and ciliary body melanoma study: a survey by the European Ophthalmic Oncology Group. Ophthalmology.

[30] Fry MV, Augsburger JJ, Correa ZM (2019). Clinical features, metastasis, and survival in patients younger than 21 years with posterior uveal melanoma. JAMA Ophthalmol.

[31] Jouhi S, Jager MJ, de Geus SJR, Desjardins L, Eide NA, Grange JD (2019). The small fatal choroidal melanoma study. a survey by the European Ophthalmic Oncology Group. Am J Ophthalmol.

[32] Afshar AR, Allen G, Damato BE (2018). The patient’s experience of ocular melanoma in the US: a survey of the Ocular Melanoma Foundation. Ocul Oncol Pathol.

[33] NHS_Oxfordshire_Clinical_Commissioning_Group. Fast Track Pathway Patient Information Leaflet. 2017 [Available from: https://www.oxfordshireccg.nhs.uk/professional-resources/documents/clinical-guidelines/cancer/fast-track-pathway-patient-information-leaflet.pdf.

[34] The_College_of_Optometrists. Pigmented fundus lesions. 2020 [Available from: https://www.college-optometrists.org/guidance/clinical-management-guidelines/pigmented-fundus-lesions.html.

[35] Royal_College_of_Ophthalmologists. Referral pathways for adult ocular tumours 2020 [Available from: https://www.rcophth.ac.uk/wp-content/uploads/2020/07/Referral-Pathways-for-Adult-Ocular-Tumours-May-2020.pdf.

[36] NHS_Interim_Management_and_Support-Intensive_Support_Team_(Cancer). Delivering Cancer Waiting Times: A good practice Guide 2014 [Available from: https://www.england.nhs.uk/wp-content/uploads/2015/03/delivering-cancer-wait-times.pdf.

[37] Oxford_Eye_Hospital. Ocular MOLES Service - Guidelines. https://www.ouh.nhs.uk/eye-hospital/work/ocular-moles-guidelines.aspx 2020 [Available from: https://www.ouh.nhs.uk/eye-hospital/work/ocular-moles-guidelines.aspx.

[38] Moorfields_Eye_Hospital_NHS_Foundation_Trust. Ocular Oncology (Eye tumours) [Available from: https://www.moorfields.nhs.uk/service/ocular-oncology-eye-tumours.

[39] Roelofs KA, O’Day R, Harby LA, Arora AK, Cohen VML, Sagoo MS, et al. The MOLES System for Planning Management of Melanocytic Choroidal Tumors: Is It Safe? Cancers (Basel). 2020;12:1311. 10.3390/cancers12051311 PMC7281649 32455720

[40] Shields CL, Dalvin LA, Ancona-Lezama D, Yu MD, Di Nicola M, Williams BK, Jr., et al. Choroidal nevus imaging features in 3,806 cases and risk factors for transformation into melanoma in 2,355 cases: The 2020 Taylor R. Smith and Victor T. Curtin Lecture. Retina. 2019;39:1840–51. 10.1097/IAE.0000000000002440 30608349

[41] Shields CL, Cater J, Shields JA, Singh AD, Santos MC, Carvalho C (2000). Combination of clinical factors predictive of growth of small choroidal melanocytic tumors. Arch Ophthalmol.

[42] Shields CL, Furuta M, Berman EL, Zahler JD, Hoberman DM, Dinh DH (2009). Choroidal nevus transformation into melanoma: analysis of 2514 consecutive cases. Arch Ophthalmol.

[43] Damato B. The MOLES acronym, scoring system and protocol for managing patients with melanocytic choroidal tumours 2020 [(Online only)]. Available from: https://www.college-optometrists.org/podcasts/oip-moles-algorithm-scoring-system-for-managing-pa. 10.1038/s41433-022-02143-x PMC9244298 35764877

[44] Flanagan JP, O’Day RF, Roelofs KA, McGuinness MB, van Wijngaarden P, Damato BE The MOLES system to guide the management of melanocytic choroidal tumours: can optometrists apply it? Clin Exp Optom. 2022:1–5. 10.1080/08164622.2022.2029685. Epub ahead of print. 10.1080/08164622.2022.2029685 35156536

[45] Shields CL, Lally SE, Dalvin LA, Sagoo MS, Pellegrini M, Kaliki S (2021). White paper on ophthalmic imaging for choroidal nevus identification and transformation into melanoma. Transl Vis Sci Technol.

